# Assessing protein similarity with Gene Ontology and its use in subnuclear localization prediction

**DOI:** 10.1186/1471-2105-7-491

**Published:** 2006-11-07

**Authors:** Zhengdeng Lei, Yang Dai

**Affiliations:** 1 Department of Bioengineering (MC063), University of Illinois at Chicago, 851 South Morgan Street, Chicago, IL 60607, USA

## Abstract

**Background:**

The accomplishment of the various genome sequencing projects resulted in accumulation of massive amount of gene sequence information. This calls for a large-scale computational method for predicting protein localization from sequence. The protein localization can provide valuable information about its molecular function, as well as the biological pathway in which it participates. The prediction of localization of a protein at subnuclear level is a challenging task. In our previous work we proposed an SVM-based system using protein sequence information for this prediction task. In this work, we assess protein similarity with Gene Ontology (GO) and then improve the performance of the system by adding a module of nearest neighbor classifier using a similarity measure derived from the GO annotation terms for protein sequences.

**Results:**

The performance of the new system proposed here was compared with our previous system using a set of proteins resided within 6 localizations collected from the Nuclear Protein Database (NPD). The overall MCC (accuracy) is elevated from 0.284 (50.0%) to 0.519 (66.5%) for single-localization proteins in leave-one-out cross-validation; and from 0.420 (65.2%) to 0.541 (65.2%) for an independent set of multi-localization proteins. The new system is available at .

**Conclusion:**

The prediction of protein subnuclear localizations can be largely influenced by various definitions of similarity for a pair of proteins based on different similarity measures of GO terms. Using the sum of similarity scores over the matched GO term pairs for two proteins as the similarity definition produced the best predictive outcome. Substantial improvement in predicting protein subnuclear localizations has been achieved by combining Gene Ontology with sequence information.

## Background

With the completion of genomic sequencing projects, the need for automated prediction of protein subcellular or subnuclear localizations becomes increasingly important. The localization of a protein can provide valuable information about its molecular function, as well as the biological pathway in which it participates [1,2]. The bulk of past work has focused on protein subcellular localizations [3-15], and has achieved high accuracy. However, the prediction of protein localization at subnuclear level is far more challenging. We have developed the first SVM-based system using protein sequence information for this task with considerable predictive accuracy [16]. In this work, we attempted to improve the performance of the system through the incorporation of information obtained from Gene Ontology (GO).

GO has been developed to help manage the overwhelming mass of current biological data that are difficult to tie together into a cohesive whole from a computational perspective [17,18]. It has become a *de facto *standard tool to annotate gene products for various databases. GO is a controlled vocabulary of terms split into three related ontologies consisting of Molecular Function (MF), Biological Processes (BP) and Cellular Components (CC). Molecular function describes activities, such as catalytic or binding activities, at the molecular level. Molecular functions generally correspond to activities that can be performed by individual gene products, but some activities are performed by assembled complexes of gene products. A biological process is series of events accomplished by one or more ordered assemblies of molecular functions. A cellular component is a component of a cell, but with the proviso that it is part of some larger object such as an anatomical structure, a gene product group. A gene product might be associated with or located in one or more cellular components [17]. It is active in one or more biological processes, during which it performs one or more molecular functions.

Each category of GO terms is structured as a directed acyclic graph (DAG). Currently there are over 20,000 GO terms [18]. The relationships between GO terms have been extensively explored and applied to various biological problems, such as search for genes with similar function. One of the key problems in these applications is how to define similarity between two GO terms. Lord *et al*. [19,20] proposed a measure based on information content for the semantic similarity of GO terms. They revealed that the semantic similarity is correlated with the protein sequence similarity and this correlation is more marked in Molecular Functional annotation. However, their definition of similarity measure relies on a particular database, e.g. SWISS-PROT. Zhang *et al*. [21] used a recursive procedure to define a statistical measure D-value (distribution value) for each GO term in the GO DAG to avoid the dependency on a single annotation database, and developed a gene functional similarity search tool. Gentleman [22] proposed two measures based on graph similarity: simUI and simLP. The former is the ratio of the number of common nodes in the two graphs reduced from the GO DAG and the number of nodes in their union. The latter is defined as the depth of the longest shared path from the root node. Wu *et al*. [23] predicted functional modules encoded in microbial genomes using a similarity measure similar to simLP.

Although the semantic similarity between two GO terms has been extensively investigated, how to define similarity between two gene products based on GO annotations for a specific application remains unclear. Suppose that each gene product is annotated by a set of GO terms. Each GO term from one set will be paired with all GO terms in the other set. There are three general ways of defining similarity for two gene products from those GO term pairs: (1) to take the maximum value from the similarity scores of GO term pairs [23,24], (2) to take average over all the similarity scores of GO term pairs [19,20], and (3) to count the number of identical GO terms in the two GO term sets [9,25]. We are particularly interested in the identification of an appropriate definition of similarity for proteins for the prediction of protein subnuclear localization. To do so, it is necessary to investigate the effect of various combinations of different measures of GO term similarity and different similarity measures of a pair of proteins on the predictive performance. This evaluation was carried out through our new predictive system expanded from the previous SVM module [16] with the addition of a nearest neighbor classification module, which was constructed based on a similarity definition between a pair of proteins.

## Results

### Dataset

To provide a valid comparison with our previous system, the same dataset as in [16] was used for evaluation of the new system. The dataset was extracted from the Nuclear Protein Database (NPD) [26] using a Perl script. The NPD is a curated database that stores information on more than 2000 vertebrate proteins, chiefly from human and mouse, which are reported in the literature to be localized in the cell nucleus. Since certain proteins are associated with more than one compartment, a test dataset consisting of proteins with multiple localizations was extracted. These proteins have the same SwissProt or Entrez Protein accession numbers although localized in different compartments. This preparative procedure resulted in 92 proteins that are localized within the six compartments described below. The majority is localized in 2 compartments and the remaining portion is localized in 3 or 4 compartments. After excluding the multi-localization proteins, a non-redundant dataset was further constructed by PROSET [27] to ensure low sequence identity (<50%). In order to have sufficient number of proteins for training and testing, only six localizations were selected for evaluation. These are PML BODY (38), Nuclear Lamina (55), Nuclear Splicing Speckles (56), Chromatin (61), Nucleoplasm (75), and Nucleolus (219). Each of these proteins has a single localization and the total number is 504. The 92 multi-localization proteins are not included in the set of 504 single-localization proteins for the leave-one-out cross-validation (LOOCV). Therefore, the multi-localization dataset is an independent testing set. The summary of the datasets is presented in Table 1.

**Table 1:** The summary of the nuclear proteins

Class label	Compartment	Number of sequences
1	PML BODY	38
2	Nuclear Lamina	55
3	Nuclear Splicing Speckles	56
4	Chromatin	61
5	Nucleoplasm	75
6	Nucleolus	219
-	Mutiple localizations	92

### Predictive system and evaluation criteria

Given a test protein with GO annotations, the similarity scores between this protein and all the other proteins in the training set are calculated from the similarity scores of GO term pairs (see Methods). The protein with the highest similarity score is designated as the nearest neighbor of the testing protein and its class label will be assigned to the test protein. If multiple proteins in various localizations attain the same highest score or the test protein does not have GO annotation, then the test protein will be assigned as "unpredicted". The unpredicted proteins will be passed on to the SVM module, which uses sequence information [16], for a full coverage of prediction.

Since the numbers of proteins for the six localizations are unbalanced, the Matthew's correlation coefficient (MCC) was employed for the optimization of parameters and evaluation of performance [28]. The overall accuracy for the multi-class classification proposed by Rost [29] was also used for the evaluation of our system. Definitions of the MCC and overall accuracy are detailed in Methods section.

### Comparison of various similarity measures for GO term pairs

Three different similarity measures for GO term pairs were compared: (1) Lord's method [20], (2) SimLP as described in Bioconductor [22], and (3) Exact Match. For Lord's method, the GO term frequencies were extracted based on UniProtKB/Swiss-Prot [30]. For a GO term pair, Exact Match defines the similarity score as 1 if the two GO terms are identical, 0 otherwise. SUM_Match was utilized to compute the similarity score between two proteins from similarity scores of GO term pairs. It takes the sum of similarity sores for all matched GO terms from two proteins. Note that the SUM_Match score is equivalent to the inner product of two GO term vectors if Exact Match is used for GO term similarity (see Methods for details). As shown in Table 2, no significant difference in performance can be observed for these three similarity measures of GO term pairs. Surprisingly, the Exact Match method, which does not utilize any DAG structure of GO, achieved competitive performance in comparison with the other two methods.

**Table 2:** Predictive results obtained by using different similarity measures for GO term pairs

Semantic similarity method	Lord	SimLP	Exact Match
Compartment	MCC (Accuracy %)		

PML BODY	0.223 (31.6)	0.253 (34.2)	0.250 (31.6)
Nuclear Lamina	0.579 (60.0)	0.578 (63.6)	0.578 (63.6)
Nuclear Splicing Speckles	0.598 (66.1)	0.607 (62.5)	0.63 (62.5)
Chromatin	0.511 (59.0)	0.518 (60.7)	0.509 (57.4)
Nucleoplasm	0.411 (50.7)	0.504 (56.0)	0.483 (54.7)
Nucleolus	0.615 (75.3)	0.656 (79.0)	0.642 (80.8)
Overall for Single-localization	**0.489 (63.7)**	**0.519 (66.5)**	**0.515(66.5)**

(Based on SUM_Match: The similarity of two proteins is defined as the sum of similarity scores over all matched GO term pairs.)

### Comparison of various similarity definitions for proteins

Very few studies have focused on exploring similarity definition of proteins based on GO terms. Two simple ways are usually employed in defining the similarity between two proteins annotated by GO terms. One is to take the maximum value from the similarity scores of GO term pairs. The other is to take average over all the similarity scores of GO term pairs. However, the above two methods produced poor results especially when the proteins were annotated by many GO terms for the prediction of protein subnuclear localization. Consequently, an extensive investigation on various similarity definitions obtained from similarity scores of GO terms was warranted. As shown in Table 3, similarity definition has profound impact on the quality of prediction. The overall accuracy ranges from 27.0% to 66.5% and overall MCC ranges from 0.141 to 0.519 for proteins with single-location. It seems that the use of the sum of similarity scores over the matched GO term pairs for two proteins as the similarity definition produces the best predictive outcome for this prediction task.

**Table 3:** Predictive results obtained by using various similarity definitions for proteins

Similarity Definition	MAX	AVG	SUM	AVG_BestPairs
Compartment	MCC (Accuracy %)			

PML BODY	0.189 (28.9)	0.153 (34.2)	0.129 (76.3)	-0.031 (0.0)
Nuclear Lamina	0.344 (45.5)	0.535 (63.6)	0.455 (45.5)	0.315 (61.8)
Nuclear Splicing Speckles	0.377 (35.7)	0.251 (71.4)	0.289 (33.9)	0.013 (12.5)
Chromatin	0.236 (19.7)	0.218 (16.4)	0.112 (4.9)	0.142 (8.2)
Nucleoplasm	0.272 (29.3)	0.039 (9.3)	-0.079 (4.0)	0.118 (6.7)
Nucleolus	0.367 (75.8)	0.431 (44.7)	0.214 (26.0)	0.289 (75.3)
Overall for Single-localization	**0.298 (50.8)**	**0.271 (40.3)**	**0.187 (27.0)**	**0.141 (42.9)**

Similarity Definition	SUM_BestPairs	AVG_Match	SUM_Match	MAX_Match

Compartment	MCC (Accuracy %)			

PML BODY	0.242 (44.7)	0.187 (34.2)	0.253 (34.2)	0.211 (31.6)
Nuclear Lamina	0.53 (67.3)	0.586 (60.0)	0.578 (63.6)	0.344 (45.5)
Nuclear Splicing Speckles	0.438 (46.4)	0.397 (66.1)	0.607 (62.5)	0.487 (46.4)
Chromatin	0.325 (36.1)	0.467 (45.9)	0.518 (60.7)	0.263 (21.3)
Nucleoplasm	0.284 (36.0)	0.332 (32.0)	0.504 (56.0)	0.298 (32.0)
Nucleolus	0.512 (66.7)	0.615 (72.6)	0.656 (79.0)	0.407 (76.7)
Overall for Single-localization	**0.388 (54.6)**	**0.431 (58.3)**	**0.519 (66.5)**	**0.335 (53.2)**

(Based on SimLP: The GO term similarity is defined on the longest path shared by two GO terms [22].)

### Effect of using GO terms from homologs

Lord *et al*. [20] reported a problem that many GO term pairs have identical similarity values. This problem stems from two sources: (1) proteins are represented by relatively small number of GO terms; (2) the similarity measure considers only the information content *p*_*ms *_(probability of the minimum subsumer) of shared parents of the query terms, meaning that the semantic distances of many different GO term pairs are identical. In order to alleviate this problem, GO terms of homologs retrieved by BLAST were used for the representation of a query protein. The parameter E-value in BLAST is crucial for the quality of homologs, as well as the number of candidate homologs. If E-value is too large, then homologs of low quality may be retrieved. On the other hand, if E-value is too small, then the number of candidate homologs retrieved becomes small. We tested the following E-value parameters: 10^0^, 10^-1^, 10^-2^, ..., 10^-10^, 10^-15^, 10^-20^, 10^-30^, 10^-50^, 10^-100^, 10^-200^, and found that E-value = 10^-9 ^was a good trade-off value. Even with this threshold the BLAST could retrieve different numbers of hits for different query proteins. We found that up to 5 homologs were suitable to represent the query protein (see Table 4).

**Table 4:** Results obtained by using different numbers of homolog(s)

Number of homlogs (up to n)	n = 1	n = 5
Compartment	MCC (Accuracy %)	

PML BODY	0.262 (39.5)	0.253 (34.2)
Nuclear Lamina	0.395 (43.6)	0.578 (63.6)
Nuclear Splicing Speckles	0.566 (57.1)	0.607 (62.5)
Chromatin	0.474 (47.5)	0.518 (60.7)
Nucleoplasm	0.457 (53.3)	0.504 (56.0)
Nucleolus	0.606 (795.)	0.656 (79.0)
Overall for Single-localization	**0.460 (62.3)**	**0.519 (66.5)**

### Predictive performance of the new system

As demonstrated before, the predictive outcome is greatly influenced by the ways of combining similarity scores of GO term pairs to give the similarity between two proteins. With the appropriate similarity definition, the performance of the current system can be significantly better than that of the previous SVM system. As seen in Table 5, the overall MCC (accuracy) is elevated from 0.284 to 0.519 (50.0% to 66.5%) for single-localization proteins in the leave-one-out cross-validation; and from 0.420 to 0.541 (65.2%, no change in accuracy) for an independent set of multi-localization proteins. More specifically, 401 (281 true predictions and 120 false predictions) out of 504 proteins were predicted by the GO module in the LOOCV, and the remaining 103 were passed on to the SVM module. For the independent test set of proteins with multi-localizations, 82 (55 true predictions and 27 false predictions) out of 92 proteins were predicted by the GO module, and the remaining 10 were passed on to the SVM module.

**Table 5:** Results obtained from the previous and current systems

Method	AA (ver1)	GO-AA (ver2)
Compartment	MCC (Accuracy %)	

PML BODY	0.172 (29.0)	0.253 (34.2)
Nuclear Lamina	0.338 (43.6)	0.578 (63.6)
Nuclear Splicing Speckles	0.363(35.7)	0.607 (62.5)
Chromatin	0.260 (19.7)	0.518 (60.7)
Nucleoplasm	0.206 (22.7)	0.504 (56.0)
Nucleolus	0.367 (76.7)	0.656 (79.0)
Overall for Single-localization	**0.284 (50.0)**	**0.519 (66.5)**
Multi-localization	**0.420 (65.2)**	**0.541 (65.2)**

It also should be noted that our system currently is designed to predict only one localization. In fact, the results shown for the proteins with multiple localizations is somewhat overestimated, as the prediction is considered correct if any one of localizations of a protein is correctly predicted.

## Discussion

GO terms have been used in the prediction of protein subcellular localization [9,25]. The similarity of two proteins was defined as the number of the exactly shared GO terms from the two proteins, or equally defined as the inner product of GO term vectors representing the two proteins (see Methods). The inner product of two GO term vectors can be considered as a special case of the similarity definition SUM_Match for two proteins used in this work. SUM_Match is essentially a weighted sum of the matched GO term pairs, where the weight is the depth of the term if SimLP is the GO term similarity; while the inner product weights uniformly 1 for all matched GO term pairs. Consequently, the more specific the two matched GO terms is, the greater the weight is; and the higher the contribution to the similarity is.

It seems that the inclusion of similarity scores of all GO term pairs is in general not a good strategy for the definition of similarity between two protein sequences. The same conclusion can be drawn for the use of scores of all best GO term pairs (see Methods). The reason may be considered as follows. If two GO terms are remotely related, but sharing a common ancestor, they still have a positive score which contributes to the similarity of two proteins. However, the similarity for protein pairs based on the matched GO terms has zero contribution from those unmatched GO terms. It seems that the unmatched terms add noise to the data and thus weaken the discriminative ability of the nearest neighbour module in our system. In our study, the best performance was attained when the similarity measure of two protein sequences is defined as SUM_Match. The similarity scores of ~20,000 matched GO term pairs can be pre-computed and stored in a hash table to effectively reduce the computation time.

A question that needs to be clarified in the GO-based approach is whether the prediction accuracy could be artificially inflated if the proteins in training or testing sets have their specific subnuclear class annotated in GO. We examined this issue as follows. In this study, there are six GO terms associated with the subnuclear compartments: PML body (GO:0016605), Nuclear lamina (GO:0005652), Nuclear speck (GO:0016607, with synonyms Nuclear speckle, Splicing speckle), Chromatin (GO:0000785), Nucleoplasm (GO:0005654), and Nucleolus (GO:0005730). All proteins annotated by any of the above six GO terms are listed in the supplementary file [see Additional file 1]. It is observed that relatively large number of proteins are correctly annotated only in two localizations: Chromatin and Nucleolus and that some proteins are mis-annotated for their subnuclear compartments. Most of them are mistakenly labelled as Nucleoplasm (GO:0005654).

To assess if these specific GO terms are influential in the prediction, the performance of the GO module was compared before and after the removal of the six GO terms from the annotation list. As shown in Table 6, the accuracies for the compartments Nuclear Lamina, Chromatin and Nucleolus decreased slightly, and those for the compartments Nuclear Splicing Speckles and Nucleoplasm increased slightly, and there is no change for the compartment PML body. The role of the GO terms of subnuclear compartments appears to be not decisive in the identification of the subnuclear compartment of a protein. Rather, the information of the overall annotated GO terms, that is, the similarity of two proteins defined from the GO term pairs is more important.

**Table 6:** Results obtained with and without the use of the six GO terms related to subnuclear compartments.

GO Module with BLAST homologs	With the subnuclear compartment GO terms	without the subnuclear compartment GO terms
Compartment	MCC (Accuracy %)	

PML BODY	0.291 (40.0)	0.290 (40.0)
Nuclear Lamina	0.626 (67.4)	0.609 (65.1)
Nuclear Splicing Speckles	0.657 (70.0)	0.640 (73.7)
Chromatin	0.544 (63.5)	0.543 (61.5)
Nucleoplasm	0.543 (58.5)	0.548 (60.0)
Nucleolus	0.744 (82.5)	0.723 (80.1)
Overall for Single-localization	**0.568 (70.1)**	**0.559 (69.2)**
Number of proteins predicted by the GO module	**401 out of 504**	**399 out of 504**

The incorporation of the GO module has substantially improved the system performance. However, the module still makes relatively high number of incorrect predictions. This error can not be corrected by the next SVM module. Therefore, it would be desirable if the system can integrate the outcomes from two modules whenever two predictions are available. We are investigating the possibility on this aspect.

Our system can be combined with other subcellular localization predictors, e.g. WoLF PSORT [32], PA-SUB [33] and pTARGET [34], for genome scale prediction of protein localizations. Our system can take a list of predicted nuclear proteins obtained from the subcellular localization predictors and make a refined prediction at the subnulear level.

## Conclusion

Gene Ontology terms have been effectively incorporated into our previous SVM-based system for the prediction of protein subnuclear localization with the use of a nearest neighbour classification module. The improvement on performance of the new system is substantial. Various similarity definitions for a pair of proteins from different similarity measures of GO terms have been examined for their effect on prediction. The use of the sum of similarity scores over the matched GO term pairs for two proteins as the similarity definition produced the best predictive outcome in our study. The extensive investigation conducted in this work may provide some guidance on the determination of similarity definition for protein pairs based on GO terms in other applications.

## Methods

### Retrieval of GO terms

Given a protein sequence, we first BLASTed it against the Swiss-Prot database with a threshold E-value = 10^-9^. We selected up to 5 homologs, and submitted the Swiss-Prot accession numbers of the homologs to the QuickGO server [31] for the retrieval of predicted GO terms. The retrieved GO terms were used to represent the given protein.

### Definitions of similarity between two GO terms

First we define the depth for each GO term as follows.

Depth(*g*_*i*_) = the distance of the longest path from GO term *g*_*i *_to the root of Gene_Ontology, i.e., GO:0003673.

**Figure 1:**
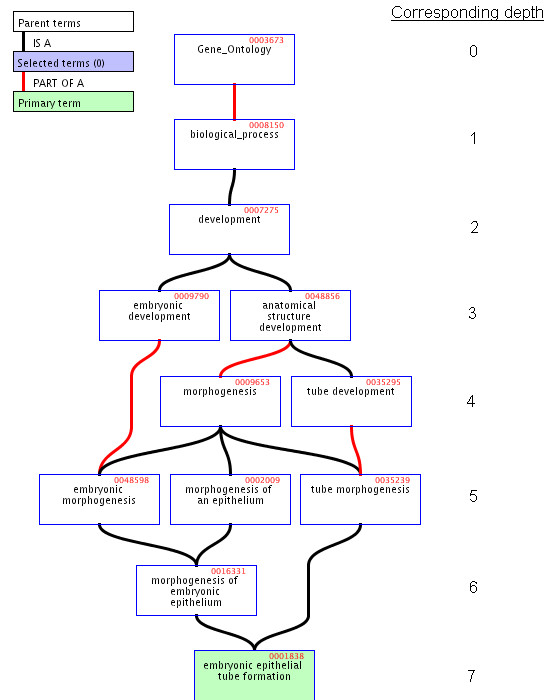
Depth of GO terms.

Fig. 1 shows an example of some GO depths, e.g. Depth(GO:0001838) = 7.

The similarity of two GO terms *g*_1 _and g_2_can be defined as the depth of their most recent common ancestor (MRCA):

Sim_GO(g1,g2)=maxgc∈P(g1,g2){Depth(gc)}.     (1)
 MathType@MTEF@5@5@+=feaafiart1ev1aaatCvAUfKttLearuWrP9MDH5MBPbIqV92AaeXatLxBI9gBaebbnrfifHhDYfgasaacH8akY=wiFfYdH8Gipec8Eeeu0xXdbba9frFj0=OqFfea0dXdd9vqai=hGuQ8kuc9pgc9s8qqaq=dirpe0xb9q8qiLsFr0=vr0=vr0dc8meaabaqaciaacaGaaeqabaqabeGadaaakeaacqWGtbWucqWGPbqAcqWGTbqBcqGGFbWxcqWGhbWrcqWGpbWtcqGGOaakcqWGNbWzdaWgaaWcbaGaemymaedabeaakiabcYcaSiabdEgaNnaaBaaaleaacqWGYaGmaeqaaOGaeiykaKIaeyypa0ZaaCbeaeaacqqGTbqBcqqGHbqycqqG4baEaSqaaiabbEgaNnaaBaaameaacqqGJbWyaeqaaSGaeyicI4SaemiuaaLaeiikaGIaem4zaC2aaSbaaWqaaiabdgdaXaqabaWccqGGSaalcqWGNbWzdaWgaaadbaGaemOmaidabeaaliabcMcaPaqabaGccqGG7bWEcqWGebarcqWGLbqzcqWGWbaCcqWG0baDcqWGObaAcqGGOaakcqWGNbWzdaWgaaWcbaGaem4yamgabeaakiabcMcaPiabc2ha9jabc6caUiaaxMaacaWLjaWaaeWaaeaacqaIXaqmaiaawIcacaGLPaaaaaa@60E0@

where *P*(*g*_1_*, g*_2_) is the set of ancestral GO terms shared by both *g*_1 _and *g*_2 _including themselves. When *g*_1 _= *g*_2_, *Depth*(*g*_*c*_) = *Depth*(*g*_1_) = *Depth*(*g*_2_). For two GO terms from different ontologies (MF, BP, CC), their MRCA is the root GO:0003673, whose depth is zero. That means that there is no similarity between two GO terms from different ontologies.

The GO term similarity described here is the same as the method simLP implemented by Gentleman [22] in Bioconductor.

### Definitions of similarity between two protein sequences

Consider two proteins that are represented respectively by the sets of GO terms *G*_1 _and *G*_2_. The similarity Sim_Pro between the two proteins can be defined as a function of Sim_GO.

For example, consider protein A (Entrez protein accession number: CAC84554) and protein B (SwissProt accession number:P46055), annotated by 3 GO terms (GO:0005488; GO:0005515; GO:0006412) and 4 GO terms (GO:0005737; GO:0006412; GO:0006415; GO:0016149), respectively. The simLP score for each GO term pair is listed in Table 7. The following 8 functions of combining similarity scores of GO term pairs were examined in this work:

**Table 7:** The simLP scores for GO term pairs

	GO: 0005737	GO: 0006412	GO: 0006415	GO:0016149
GO: 0005488	0	0	0	2
GO: 0005515	0	0	0	2
GO: 0006412	0	7	7	0

(a) MAX: take the maximum similarity score from the similarity scores of all pairs of GO terms. Sim_Pro = 7.

(b) AVG: take the average similarity score over all pairs of GO terms. Sim_Pro = (7+7+2+2)/12 = 1.5.

(c) SUM: take the sum over all pairs of GO terms, Sim_Pro = 7+7+2+2 = 18.

(d) MAX_Match: same as (a), except that only the matched GO term pairs are considered, e.g. GO:0006412. Sim_Pro = 7.

(e) AVG_Match: same as (b), except that only the matched GO term pairs are considered, e.g. GO:0006412. Sim_Pro = 7/1 = 7.

(f) SUM_Match: same as (c), except that only the matched GO term pairs are considered, e.g. GO:0006412. Sim_Pro = 7.

(g) AVG_BestPairs: Average similarity between the best paired GO terms calculated with the following pseudo codes:

NumofBestPairs ← min {|*G_1_*|, |*G_2_*|}

Sim_Pro ← 0

While (|*G_1_*|>0 and |*G_2_*|>0)

Max_sim_GO ← max{Sim_GO(g_*i*_, g_*j*_)}, *g*_*i *_∈ *G*_1_, *g*_*j *_∈ *G*_2_

Sim_Pro ← Sim_Pro + Max_sim_GO

Delete g_*i *_from G_1_, and g_*j *_from *G*_2 _

End while

Sim_Pro ← Sim_Pro/NumofBestPairs

Sim_Pro = (7+2+0)/3 = 3.

(h) SUM_BestPairs: same as (g), except that we do not divide Sim_Pro by NumofBestPairs, i.e., remove the last line in the pseudo codes in (g). Sim_Pro = 7+2+0 = 9.

In this work, the similarity Sim_Pro of two proteins employed in the final system is based on function (f) SUM_Match:

Sim_PRO(p1,p2)=∑gi=gjSim_GO(gi,gj),gi∈G1,gj∈G2     (2)
 MathType@MTEF@5@5@+=feaafiart1ev1aaatCvAUfKttLearuWrP9MDH5MBPbIqV92AaeXatLxBI9gBaebbnrfifHhDYfgasaacH8akY=wiFfYdH8Gipec8Eeeu0xXdbba9frFj0=OqFfea0dXdd9vqai=hGuQ8kuc9pgc9s8qqaq=dirpe0xb9q8qiLsFr0=vr0=vr0dc8meaabaqaciaacaGaaeqabaqabeGadaaakeaafaqabeqacaaabaGaem4uamLaemyAaKMaemyBa0Maei4xa8LaemiuaaLaemOuaiLaem4ta8KaeiikaGIaemiCaa3aaSbaaSqaaiabdgdaXaqabaGccqGGSaalcqWGWbaCdaWgaaWcbaGaemOmaidabeaakiabcMcaPiabg2da9maaqafabaGaem4uamLaemyAaKMaemyBa0Maei4xa8Laem4raCKaem4ta8KaeiikaGIaem4zaC2aaSbaaSqaaiabdMgaPbqabaGccqGGSaalcqWGNbWzdaWgaaWcbaGaemOAaOgabeaakiabcMcaPaWcbaGaem4zaC2aaSbaaWqaaiabdMgaPbqabaWccqGH9aqpcqWGNbWzdaWgaaadbaGaemOAaOgabeaaaSqab0GaeyyeIuoakiabbYcaSaqaaiabdEgaNnaaBaaaleaacqWGPbqAaeqaaOGaeyicI4Saem4raC0aaSbaaSqaaiabdgdaXaqabaGccqWGSaalcqWGGaaicqWGGaaicqWGNbWzdaWgaaWcbaGaemOAaOgabeaakiabgIGiolabdEeahnaaBaaaleaacqWGYaGmaeqaaaaakiaaxMaacaWLjaWaaeWaaeaacqaIYaGmaiaawIcacaGLPaaaaaa@6B52@

where Sim_GO is defined in (1). Alternatively, if Sim_GO is defined as a constant 1, the Sim_Pro is exactly the Inner Product of two GO vectors (see below).

### Inner product of two GO term vectors

The Inner Product of two GO term vectors has been used in previous study for the prediction of protein subcellular localization [9,25]. A vector with a length equal to the number of all appeared GO terms is prepared for a given protein. An entry is assigned a value 1 if the corresponding GO term is used for the annotation of the protein, 0 otherwise. Then each protein is represented by a binary vector. The similarity between two proteins is defined as the inner product of the two corresponding GO term vectors. Alternatively, Inner Product is the same as the total number of the matched GO terms from the annotation lists of the two proteins.

### Nearest neighbor classification

Our system includes a K-Nearest Neighbor (KNN) model. The best result was achieved with K = 1. A protein is assigned with a localization label of its nearest neighbor that has the highest similarity score Sim_Pro. If the protein does not have associated GO terms or has multiple nearest neighbors in various classes, then the second SVM module built on sequence information [16] will be called to give a prediction.

### The SVM module

In our previous work [16], we built an SVM system for prediction of protein subnuclear localizations based solely on protein sequence information. New SVM kernel functions were introduced for the measure of sequence similarity. The k-peptide vectors are first mapped by a matrix of high-scored pairs of k-peptides which are measured by BLOSUM62 scores. The kernels, measuring the similarity for sequences, are then defined on the mapped vectors. By combining these new encoding methods, a multi-class classification system for the prediction of protein subnuclear localizations was established.

### Evaluation

Since the numbers of proteins for various localizations are unbalanced, the Matthew's correlation coefficient (MCC) was employed for the optimization of parameters and evaluation of performance [28]:

MCCn=pnsn−unon(pn+un)(pn+on)(sn+un)(sn+on),
 MathType@MTEF@5@5@+=feaafiart1ev1aaatCvAUfKttLearuWrP9MDH5MBPbIqV92AaeXatLxBI9gBaebbnrfifHhDYfgasaacH8akY=wiFfYdH8Gipec8Eeeu0xXdbba9frFj0=OqFfea0dXdd9vqai=hGuQ8kuc9pgc9s8qqaq=dirpe0xb9q8qiLsFr0=vr0=vr0dc8meaabaqaciaacaGaaeqabaqabeGadaaakeaacqWGnbqtcqWGdbWqcqWGdbWqdaWgaaWcbaGaemOBa4gabeaakiabg2da9maalaaabaGaemiCaa3aaSbaaSqaaiabd6gaUbqabaGccqWGZbWCdaWgaaWcbaGaemOBa4gabeaakiabgkHiTiabdwha1naaBaaaleaacqWGUbGBaeqaaOGaem4Ba82aaSbaaSqaaiabd6gaUbqabaaakeaadaGcaaqaaiabcIcaOiabdchaWnaaBaaaleaacqWGUbGBaeqaaOGaey4kaSIaemyDau3aaSbaaSqaaiabd6gaUbqabaGccqGGPaqkcqGGOaakcqWGWbaCdaWgaaWcbaGaemOBa4gabeaakiabgUcaRiabd+gaVnaaBaaaleaacqWGUbGBaeqaaOGaeiykaKIaeiikaGIaem4Cam3aaSbaaSqaaiabd6gaUbqabaGccqGHRaWkcqWG1bqDdaWgaaWcbaGaemOBa4gabeaakiabcMcaPiabcIcaOiabdohaZnaaBaaaleaacqWGUbGBaeqaaOGaey4kaSIaem4Ba82aaSbaaSqaaiabd6gaUbqabaGccqGGPaqkaSqabaaaaOGaeiilaWcaaa@633A@

where *p*_*n *_is the number of correctly predicted proteins of the location *n*, *s*_*n *_is the number of correctly predicted proteins not in the location *n*, *u*_*n *_is the number of under-predicted proteins, and *o*_*n *_the number of over-predicted proteins.

Also, the overall accuracy for the multi-class classification proposed by Rost [29] was used for the evaluation of our system. Suppose there are *m *= *m*_1 _+ *m*_2 _+ … + *m*_*N *_test proteins, where *m*_*n *_is the number of proteins belonging to class *n*(*n *= 1,...,*N*). Suppose further that out of the proteins considered, *p*_*n *_proteins are predicted to belong to class *n*. Then *p *= *p*_1 _+ *p*_2 _+ … + *p*_*N *_is the number of correctly predicted proteins. The accuracy for class *n *is

accn=pnmn,
 MathType@MTEF@5@5@+=feaafiart1ev1aaatCvAUfKttLearuWrP9MDH5MBPbIqV92AaeXatLxBI9gBaebbnrfifHhDYfgasaacH8akY=wiFfYdH8Gipec8Eeeu0xXdbba9frFj0=OqFfea0dXdd9vqai=hGuQ8kuc9pgc9s8qqaq=dirpe0xb9q8qiLsFr0=vr0=vr0dc8meaabaqaciaacaGaaeqabaqabeGadaaakeaacqWGHbqycqWGJbWycqWGJbWydaWgaaWcbaGaemOBa4gabeaakiabg2da9maalaaabaGaemiCaa3aaSbaaSqaaiabd6gaUbqabaaakeaacqWGTbqBdaWgaaWcbaGaemOBa4gabeaaaaGccqGGSaalaaa@3A28@

and the overall accuracy, denoted by Q_acc_, is defined as

Qacc=∑n=1Naccn×mnm=∑n=1Npnm=pm.
 MathType@MTEF@5@5@+=feaafiart1ev1aaatCvAUfKttLearuWrP9MDH5MBPbIqV92AaeXatLxBI9gBaebbnrfifHhDYfgasaacH8akY=wiFfYdH8Gipec8Eeeu0xXdbba9frFj0=OqFfea0dXdd9vqai=hGuQ8kuc9pgc9s8qqaq=dirpe0xb9q8qiLsFr0=vr0=vr0dc8meaabaqaciaacaGaaeqabaqabeGadaaakeaacqWGrbqudaWgaaWcbaGaemyyaeMaem4yamMaem4yamgabeaakiabg2da9maaqahabaGaemyyaeMaem4yamMaem4yam2aaSbaaSqaaiabd6gaUbqabaGccqGHxdaTdaWcaaqaaiabd2gaTnaaBaaaleaacqWGUbGBaeqaaaGcbaGaemyBa0gaaaWcbaGaemOBa4Maeyypa0JaeGymaedabaGaemOta4eaniabggHiLdGccqGH9aqpdaaeWbqaamaalaaabaGaemiCaa3aaSbaaSqaaiabd6gaUbqabaaakeaacqWGTbqBaaGaeyypa0ZaaSaaaeaacqWGWbaCaeaacqWGTbqBaaaaleaacqWGUbGBcqGH9aqpcqaIXaqmaeaacqWGobGta0GaeyyeIuoakiabc6caUaaa@56E3@

## Availability and requirements

Project name: Subnuclear Compartments Prediction System (Version 2.0)

Project home page: 

Operating system(s): Linux

Programming language: Perl

License: None

Any restrictions to use by non-academics: None

## Authors' contributions

LZ designed the system, implemented programs and carried out the detail study. YD conceived the idea of this work, supervised project and participated in manuscript preparation. All authors have read and approved the final manuscript.

## Note

AA : SVM module based on protein sequence information

GO-AA: Combination of Gene Ontology module and sequence information module

Lord: The GO term similarity is defined on information content by Lord *et al*. [20]

SimLP: The GO term similarity is defined as the longest path shared by two GO terms [22]

Exact Match: The GO term similarity is defined as 1 if two GO terms are identical, 0 otherwise.

MAX: The similarity of two proteins is defined as the maximum of the similarity scores of all GO term pairs

AVG: The similarity of two proteins is defined as the average of the similarity scores of all GO term pairs

SUM: The similarity of two proteins is defined as the sum of similarity scores over all GO term pairs

MAX_Match: The similarity of two proteins is defined as the maximum of similarity scores of all matched GO term pairs

AVG_Match: The similarity of two proteins is defined as the average of similarity scores of all matched GO term pairs

SUM_Match: The similarity of two proteins is defined as the sum of similarity scores over all matched GO term pairs

AVG_BestPairs: The similarity of two proteins is defined as the average of similarity scores of the best paired GO terms

SUM_BestPairs: The similarity of two proteins is defined as the sum of similarity scores over all best paired GO terms

## Supplementary Material

Additional File 1 GO annotation for single-localization proteins. The data provides single-localization proteins annotated by six subnuclear compartment GO terms. Click here for file

## References

[B1] Cocco L, Manzoli L, Barnabei O, Martelli AM (2004). Significance of subnuclear localization of key players of inositol lipid cycle. Adv Enzyme Regul.

[B2] Itoh K, Brott BK, Bae GU, Ratcliffe MJ, Sokol SY (2005). Nuclear localization is required for Dishevelled function in Wnt/beta-catenin signaling. J Biol.

[B3] Nakai K, Horton P (1999). PSORT: a program for detecting sorting signals in proteins and predicting their subcellular localization. Trends Biochem Sci.

[B4] Feng ZP (2002). An overview on predicting the subcellular location of a protein. In Silico Biol.

[B5] Gardy JL, Spencer C, Wang K, Ester M, Tusnady GE, Simon I, Hua S, deFays K, Lambert C, Nakai K (2003). PSORT-B: Improving protein subcellular localization prediction for Gram-negative bacteria. Nucleic Acids Res.

[B6] Nair R, Rost B (2003). Better prediction of sub-cellular localization by combining evolutionary and structural information. Proteins.

[B7] Lu X, Zhai C, Gopalakrishnan V, Buchanan BG (2004). Automatic annotation of protein motif function with Gene Ontology terms. BMC Bioinformatics.

[B8] Tu K, Yu H, Guo Z, Li X (2004). Learnability-based further prediction of gene functions in Gene Ontology. Genomics.

[B9] Cai YD, Chou KC (2004). Predicting 22 protein localizations in budding yeast. Biochem Biophys Res Commun.

[B10] Gardy JL, Laird MR, Chen F, Rey S, Walsh CJ, Ester M, Brinkman FS (2005). PSORTb v.2.0: expanded prediction of bacterial protein subcellular localization and insights gained from comparative proteome analysis. Bioinformatics.

[B11] Bhasin M, Garg A, Raghava GP (2005). PSLpred: prediction of subcellular localization of bacterial proteins. Bioinformatics.

[B12] Sarda D, Chua GH, Li KB, Krishnan A (2005). pSLIP: SVM based protein subcellular localization prediction using multiple physicochemical properties. BMC Bioinformatics.

[B13] Wang J, Sung WK, Krishnan A, Li KB (2005). Protein subcellular localization prediction for Gram-negative bacteria using amino acid subalphabets and a combination of multiple support vector machines. BMC Bioinformatics.

[B14] Bjorklund AK, Ekman D, Light S, Frey-Skott J, Elofsson A (2005). Domain rearrangements in protein evolution. J Mol Biol.

[B15] Nair R, Rost B (2005). Mimicking cellular sorting improves prediction of subcellular localization. J Mol Biol.

[B16] Lei Z, Dai Y (2005). An SVM-based system for predicting protein subnuclear localizations. BMC Bioinformatics.

[B17] Harris MA, Clark J, Ireland A, Lomax J, Ashburner M, Foulger R, Eilbeck K, Lewis S, Marshall B, Mungall C (2004). The Gene Ontology (GO) database and informatics resource. Nucleic Acids Res.

[B18] http://www.geneontology.org/.

[B19] Lord PW, Stevens RD, Brass A, Goble CA (2003). Semantic similarity measures as tools for exploring the gene ontology. Pac Symp Biocomput.

[B20] Lord PW, Stevens RD, Brass A, Goble CA (2003). Investigating semantic similarity measures across the Gene Ontology: the relationship between sequence and annotation. Bioinformatics.

[B21] Zhang P, Zhang J, Sheng H, Russo JJ, Osborne B, Buetow K (2006). Gene functional similarity search tool (GFSST). BMC Bioinformatics.

[B22] Gentleman R (2005). Visualizing and Distances Using GO. http://www.bioconductor.org/repository/devel/vignette/GOvis.pdf.

[B23] Wu H, Su Z, Mao F, Olman V, Xu Y (2005). Prediction of functional modules based on comparative genome analysis and Gene Ontology application. Nucleic Acids Res.

[B24] Wu X, Zhu L, Guo J, Zhang DY, Lin K (2006). Prediction of yeast protein-protein interaction network: insights from the Gene Ontology and annotations. Nucleic Acids Res.

[B25] Chou KC, Cai YD (2005). Predicting protein localization in budding yeast. Bioinformatics.

[B26] Dellaire G, Farrall R, Bickmore WA (2003). The Nuclear Protein Database (NPD): sub-nuclear localisation and functional annotation of the nuclear proteome. Nucleic Acids Res.

[B27] Brendel V (1992). PROSET – a fast procedure to create non-redundant sets of protein sequences. Mathl Comput Modelling.

[B28] Matthews BW (1975). Comparison of the predicted and observed secondary structure of T4 phage lysozyme. Biochim Biophys Acta.

[B29] Rost B, Sander C (1993). Prediction of protein secondary structure at better than 70% accuracy. J Mol Biol.

[B30] http://www.pir.uniprot.org/database/download.shtml.

[B31] http://www.ebi.ac.uk/ego/.

[B32] http://wolfpsort.seq.cbrc.jp/.

[B33] http://www.cs.ualberta.ca/~bioinfo/PA/Sub/.

[B34] http://bioinformatics.albany.edu/~ptarget.

